# Harmonisation of switched memory B cell analysis for improved CVID diagnosis and classification

**DOI:** 10.3389/fimmu.2025.1726673

**Published:** 2026-01-13

**Authors:** Malicorne Buysse, Jana Neirinck, Ciel De Vriendt, Filomeen Haerynck, Martín Pérez-Andrés, Alberto Orfao, Mattias Hofmans, Carolien Bonroy

**Affiliations:** 1 Department of Laboratory Medicine, Ghent University Hospital, Ghent, Belgium; 2 Department of Haematology, Ghent University Hospital, Ghent, Belgium; 3 Department of Pediatric Pulmonology and Immunology and Primary Immunodeficiency (PID) Research Laboratory, Ghent University Hospital, Ghent, Belgium; 4 Cancer Research Centre (Instituto de Biología Molecular y Celular del Cáncer (IBMCC), Cancer Research Institute of Salamanca (USAL-CSIC), Spanish Biomedical Research Centre in Cancer (CIBERONC) CB16/12/00400, Institute for Biomedical Research of Salamanca (IBSAL), Department of Medicine and Cytometry Service (NUCLEUS Research Support Platform), University of Salamanca (USAL), Salamanca, Spain; 5 Translational and Clinical Research Program, Centro de Investigación del Cáncer and Instituto de Biología Molecular Salamanca (USAL), Department of Medicine, IBSAL and Centro de Investigación Biomédica en Red de Cáncer (CIBERONC), University of Salamanca, Salamanca, Spain; 6 Department of Diagnostic Sciences, Ghent University, Ghent, Belgium

**Keywords:** ESID, EUROclass, flow cytometry (FCM), common variable immunodeficiency (CVID), EuroFlow (EF), harmonisation

Common variable immunodeficiency (CVID) is an inborn error of immunity (IEI) characterized by impaired immunoglobulin (Ig) production, in association with an increased susceptibility to infections and a diversity of non-infectious clinical manifestations, such as autoimmunity and organomegalies (1). Most CVID patients also show abnormalities in B-cell maturation patterns, mostly at the plasma cell (PC) and memory B-cell (MBC) levels, as confirmed by flow cytometric (FCM)-based immunophenotyping (2, 3).

Since 1998, human MBC have been immunophenotypically defined as CD27^+^(i.e., classical MBC) (4, 5). This cell surface receptor protein is a member of the TNF-receptor family and was considered the preferred marker to distinguish MBC from antigen-inexperienced (naïve) B cells (IgD^+^) based on the established correlation between its expression and the somatic hypermutation status (SHM) of Ig genes (2, 6, 7). Today, it is recognized that approximately 1-5% of human peripheral blood memory B-cells in healthy adults lack CD27 expression on their surface membrane (6, 7). Some studies described increased frequencies of double-negative IgD^-^/CD27^-^ (DN) B cells in peripheral blood of healthy controls aged > 60 years, compared to younger adults (8), although this age-related increase in DN B cells was not consistently confirmed across studies (9) and their functional role remains to be fully elucidated. Published data suggest that DN B cells are possibly heterogeneous as some authors regarded them as exhausted cells due to repeated antigenic stimulation, while others consider them as T-cell independent or primary less experienced germinal center (GC) memory B cells (8, 10). Those IgD^-^/CD27^-^ DN B cells share several characteristics with the IgD^-^/CD27^+^ switched memory B cells (sMBC), including similar telomere length and morphology. However, the level of SHM of class-switched Ig genes of IgD^-^/CD27^-^ DN B cells is somewhat lower than that of IgD^-^/CD27^+^ sMBC (6–8, 10). Thus, the definition of sMBC has progressed over time, currently recognizing sMBC as heterogeneous for CD27. This is of particular importance when sMBC are part of the diagnostic/prognostic criteria of a disease, as is the case in CVID.

Indeed, the current European Society for Immunodeficiencies (ESID) working definitions for the clinical diagnosis of CVID (published in November 2019) include low sMBC (<70% of age-matched normal values) as a supportive (but not mandatory) diagnostic criterion (11). Likewise, the well-established EUROclass classification categorizes CVID patients based on the percentage of sMBC (≤ 2%) into the SmB^-^ vs SmB^+^ groups associated with relevant differences in disease severity and their clinical phenotypes (2). However, no definitive consensus exists, nor any explicit guidance has been provided in the ESID diagnostic criteria, on how sMBC should be immunophenotypically defined, what reference values should be applied and whether ‘low’ sMBC refers to relative (%) or absolute cell counts (µL).

In this study, we investigated the impact of different sMBC definitions/tube compositions/reference values on the subclassification of CVID using two EuroFlow (EF) standardized FCM panels. Both panels are part of the EF FCM-based algorithm for the diagnostic work-up of primary immunodeficiencies (PID) of the lymphoid system (12). In the context of predominantly antibody deficiencies (PAD) including CVID, this algorithm recommends the combined use of the 8-color PID orientation tube (PIDOT) and the 12-color immunoglobulin IgH-isotype tube to allow for a detailed analysis of the MBC and plasmablast compartments in blood, enabling the identification of specific CVID subtypes (12). Compared to PIDOT, the IgH-isotype tube includes additional markers (such as CD38, CD5 and CD24, IgG1-4, IgA1-2), and both tubes are accompanied by distinct sets of tube-specific age-related reference values generated by the EuroFlow Consortium (13). More details on the antibody panels used in this study are included in **Supplementary Table S1**. Furthermore, the standardized FCM gating strategies proposed for both tubes define sMBC based on heterogeneous CD27 expression (CD27^het^) in the absence of IgM/D cell surface expression (12–14). For more details on the gating strategies of PIDOT and IgH tubes see Neirinck et al., 2022 and Blanco et al., 2019, respectively. This approach differs from the commonly used EUROclass CVID classification, in which CD27 positivity is key for the identification of MBC (2).

In a three year period (from 03/2021 to 03/2024), standardized FCM analysis was performed on fresh (<24 hours following collection) peripheral blood samples from 33 CVID patients (M/F ratio: 17/16; average age in years (min-max): 37 (12 – 75)) using the EF PIDOT and IgH-isotype tubes at the Laboratory of Hematology and Clinical Immunology of Ghent University Hospital (Ghent, Belgium) (12, 13, 15). FCM analysis was performed strictly following the EF standard operating procedures (SOPs) for bulk lysis, sample staining, instrument set-up and calibration (www.EuroFlow.org). Data were acquired on a FACSLyric flow cytometer (Beckton Dickinson, San Jose, CA) and analyzed with the Infinicyt software (version 2.0.6.b.007; BD-Cytognos S.L., Salamanca, Spain). Ethical approval for this study was obtained (EC-approval code: BC-07300).

All flow cytometry standard (fcs) data files originating from the two-tube stainings were manually analyzed in duplicate using the two different definitions for sMBC (heterogeneous expression of CD27^het^ vs. CD27^+^ expression). Relative cell counts (% on parent population) were obtained for CD27^+^ and CD27^het^ sMBC. Absolute cell counts were obtained by the dual-platform flow cytometry method using the total lymphocyte counts originating from the Sysmex XE-5000 or XN-1000 hematology analyzers (Sysmex Corporation, Kobe, Japan) (16). Patients were subsequently categorized into the SmB^+^ or SmB^-^ CVID groups using the EUROclass criterion of ≤ 2%. Differences between tube stainings and reference values were also investigated. Statistical evaluation and graphical representations were performed using SPSS (version 29.0.2.0, IBM, Armonk, NY), Rstudio (version 100 4.3.3) and Biorender.com.

**Figure 1:**
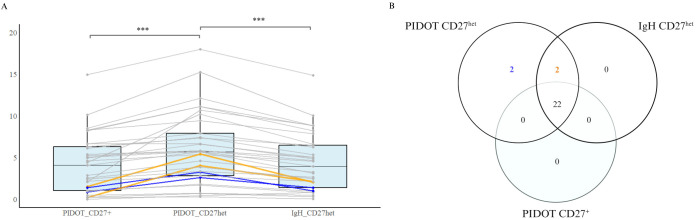
The impact of different definitions and FCM tube compositions on the identification and quantification of switched MBC (sMBC). **(A)** Paired box plots show relative cell counts (% on parent population) of sMBC, obtained via different gating strategies (CD27^+^ vs CD27^het^) and tube compositions (PIDOT vs IgH-isotype tube). (Paired sample T-test, ***p-values <0.001 were considered statistically significant). **(B)** Venn diagram representing the number of CVID patients (n = 26/33) that fulfill the SmB^+^ criterion (sMBC > 2%) according to the EUROclass classification, based on different FCM tubes and gating strategies. Discordant cases due to differences in the sMBC defining criteria are indicated in orange. The other discordant cases attributed to a combination of sMBC definition and/or tube composition are indicated in blue. Figure created with BioRender.com.

Our data shows that both differences in definition of sMBCs and the tube composition influenced the relative (**Figure 1A**) and absolute counts of sMBC (**Supplementary Figure S1**). Thus, with PIDOT, both relative (from total B cells) and absolute sMBC counts were significantly higher when using the EF gating approach (CD27^het^) versus the historical gating approach (CD27^+^) (median 5.7% vs. 4.1%, p < 0.001, median 8.3/µL vs. 4.6/µL, p <0.001; Paired t-test). Interestingly, the IgH-isotype tube yielded a significantly lower percentage of sMBC compared to PIDOT (median 4.0% vs. 5.7%, p < 0.001, median 4.7/µL vs. 8.3/µL, p <0.001), although both tubes use the same definition of CD27^het^. In part, this difference can be explained by the usage of different strategies to define and exclude plasma cells. These findings are in line with the reference values published by EF for the PIDOT and the IgH-isotype antibody panels which do not completely overlap for the sMBC population, as reference ranges are tube-specific and therefore not interchangeable between the two standardized EF PID tubes, nor can they be generalized to other FCM tubes using other markers and clones. Of note, age groups used to establish reference ranges are also not fully aligned. Together, this may yield differences in interpretation, especially in case of borderline results.

Our observations show that evolutions in definitions of sMBC (CD27^+^ vs CD27^het^) and marker choices affect the identification of sMBC. Moreover, this information is often not shared by the laboratories with the treating clinicians. This entails the risk of a mismatch between the definition of sMBC used and the classification used. To investigate the impact of this potential mismatch, we classified our 33 CVID patients in smB^+^ vs smB^-^ groups using the EUROclass criterion of ≤2% sMBC. As expected, applying the newer CD27^het^ definition of sMBC resulted in significantly more patients being classified as SmB^+^ (>2% sMBC) compared to the traditional (CD27^+^) gating strategy. This was the case for both the EF PIDOT (79% vs 67%, p < 0.001) and the EF IgH-isotype tubes (73% vs 55%, p < 0.001). Overall, this implies that 12% of CVID (n=4/33) patients in our cohort were differentially subcategorized as SmB^+^ depending on the gating strategy (see **Figure 1B**; **Supplementary Table S2**). Interestingly, in two of these cases also differences in tube composition affected the SmB interpretation.

In conclusion, we illustrated that differences in gating strategies and marker choices may (substantially) affect the identification of sMBC, as well as the subclassification of a small fraction of CVID patients, potentially impacting CVID diagnosis. In our work we mainly focused on the definition of sMBC based on CD27, but impact on the proportion may also be seen when changing the denominator for calculating sMBC (17). We therefore encourage policymakers to unambiguously define the sMBC in the CVID diagnostic and classification criteria, by specifying at least the markers and the data analysis strategy, the denominator, and ideally, also introducing age-matched reference values or age-normalized criteria instead of a single age-independent cut-off. From a laboratory perspective, we can tackle this challenge by mentioning the way sMBC are defined, and by implementing standardized EF tubes and protocols, enabling consistent and reproducible CVID classification across laboratories, ultimately leading to improved patient care.
