# Supplementary material for: Harmonisation of switched memory B cell analysis for improved CVID diagnosis and classification
Source: Front Immunol. 2026 Jan 13;16:1726673. doi: 10.3389/fimmu.2025.1726673 (PMC12835365; doi:10.3389/fimmu.2025.1726673)
Supplement: Supplementary file 1 [file DataSheet1.pdf]

Supplementary Material

Supplementary Figure

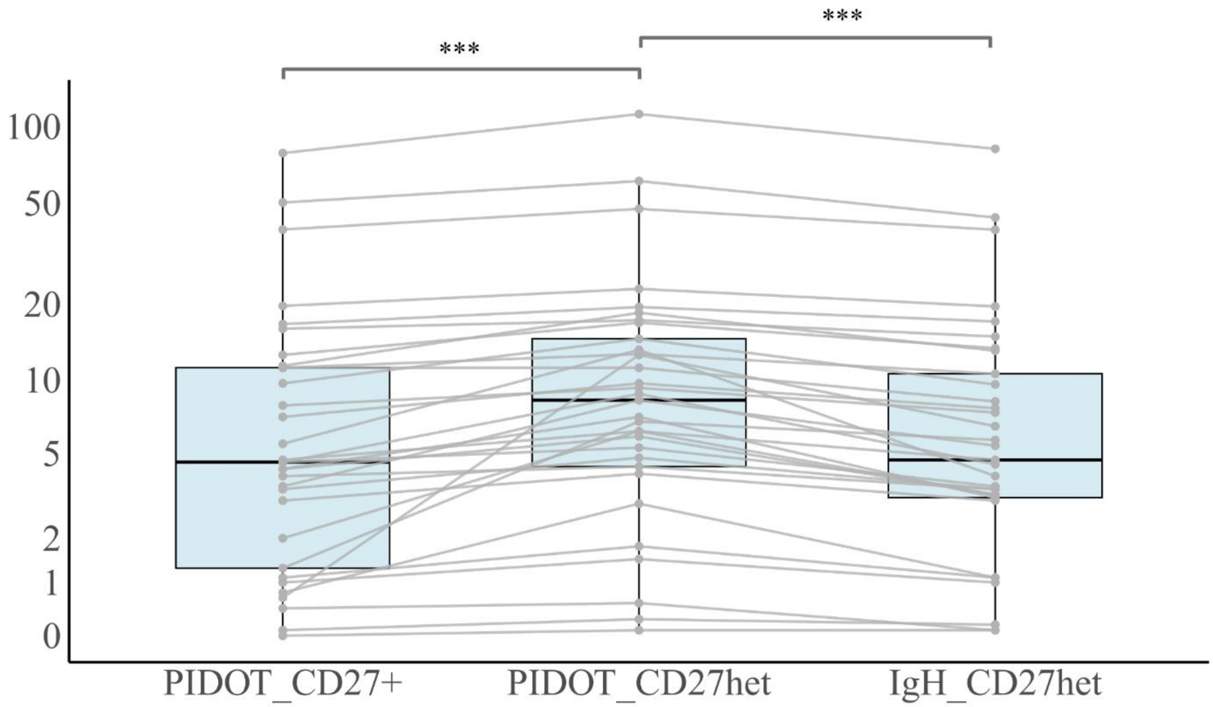

Supplementary Figure S1 The impact of different definitions and FCM tube compositions on sMBC identification. Paired box plots show absolute cell counts (/µL, expressed on a pseudolog scale) of sMBC, obtained via different gating strategies ($CD27^{+}$ vs $CD27^{het}$) and tube compositions (PIDOT vs IgH-isotype tube). Paired sample T-test, ***p-values <0.001 were considered statistically significant.

Supplementary Tables

	Markers	
**Fluorochome**	**PIDOT tube**	**IgH tube**
**FITC**	CD8+IgD	IgG2+IgG3
**PE**	CD16+CD56	IgG1+IgG2
**PerCP-Cy5.5**	CD4+IgM	IgA1+ IgA2
**PE-Cy7**	CD19+TCRγδ	CD5
**APC**	CD3	IgA1+IgG4
**APC-C750**	CD45	/
**APC-H7**	/	CD38
**APC AF700**	/	IgD
**BV421**	CD27	CD27
**BV510**	CD45RA	IgM
**BV605**	/	CD24
**BV711**	/	CD21
**BV786**	/	CD19

Supplementary Table S1 The markers and fluorochromes included in the PIDOT and IgH-isotype tube.

ID	PIDOT CD27+ (%)	Interpretation	PIDOT CD27het (%)	Interpretation	IgH CD27het (%)	Interpretation
116	**0.22**	SmB-	4.04	SmB+	2.10	SmB+
243	**1.50**	SmB-	5.40	SmB+	2.02	SmB+
58	**0.84**	SmB-	2.58	SmB+	**1.38**	SmB-
113	**1.36**	SmB-	3.24	SmB+	**0.98**	SmB-

Supplementary Table S2 Overview of the relative counts of the discrepant results (4/33) using the cut-off of 2% sMBC. Results below 2% are shown in bold, borderline results are marked in green.
